# Thyroid gland dysfunction and vitamin D receptor gene polymorphism in keratoconus

**DOI:** 10.1038/s41433-022-02172-6

**Published:** 2022-08-01

**Authors:** Eman A. Awad, Magda A. Torky, Rania M. Bassiouny, Abeer M. Khattab, Rasha R. Elzehery, Rania M. Elhelaly

**Affiliations:** 1 Department of Ophthalmology, Mansoura Ophthalmic Center, Faculty of Medicine, Mansoura University, Mansoura, Egypt; 2 Department of Clinical Pathology, Faculty of Medicine, Mansoura University, Mansoura, Egypt

**Keywords:** Gene expression, Corneal diseases

## Abstract

**Objectives:**

To detect the serum level of thyroid hormones, vitamin D and vitamin D receptors (VDR) polymorphism in keratoconus (KC) patients and to identify the association between vitamin D deficiency and thyroid dysfunction in KC.

**Methods:**

This cross sectional study included 177 KC patients with no thyroid disorders compared to 85 healthy controls with normal corneal tomography. Measurements of thyroid stimulating hormone (TSH), free triiodothyronine (FT3), free tetraiodothyronine (FT4) and serum 25-OH vitamin D were done using Enzyme linked immusoassay (ELISA test). VDR polymorphisms were tested including [Taq I (rs731236), Apa I (rs7975232) and Bsm I (rs1544410)] using polymerase chain reaction-restriction fragment length polymorphism (PCR-RFLP).

**Results:**

An increase in frequency of thyroid disorders (*P* = 0.04), decrease in serum 25(OH) vitamin D level (*P* < 0.001), Taq 1 and tt genotype (*P* < 0.001) were significantly distributed in KC patients. A significantly higher serum 25(OH) vitamin D level was reported in TT genotype, while insufficient level was more common in Tt genotype (*P* < 0.001). A deficient serum 25(OH) vitamin D level was predominant in tt genotype (*P* < 0.001). A 95% confidence interval was in TSH (1.603, 2.946), FT4 (24.145, 77.06), hypothyroidism (1.062, 67.63), insufficient (2.936, 11.643) and deficient vitamin D (5.283, 28.704) and all were significant risk factors for KC with (*P* < 0.05).

**Conclusions:**

Both thyroid disorders and low vitamin D are potential factors for KC development. Studying VDR at the molecular level provides interesting avenues for future research toward the identification of new KC cases.

## Introduction

Keratoconus (KC) is a progressive ectatic corneal disease, characterized by conical protrusion of the cornea with progressive thinning resulting in myopia, irregular astigmatism, and associated with severe visual impairment [1]. The exact pathophysiology of KC is not fully explained, many theories had been suggested based on immunological, genetic, and environmental factors [2–6]. Various inflammatory mediators (Cytokines) were linked with KC suggesting a pivotal role of inflammation in the pathogenesis, of the disease [7]. The prevalence of thyroid gland dysfunction was reported in KC patients. Thyroxin was found to cause biochemical changes in the corneal stroma as a result of thyroxin–receptor interaction [8–11]. Vitamin D receptors (VDR) are found in the cornea, lens, and retinal pigment epithelium [12, 13]. The effect of vitamin D on KC was evaluated by Shivakumar et al. [14] in their in vitro study which showed that vitamin D enhances VDR and activates autophagic lysosomal clearance in oxidatively damaged human corneal epithelial cells. An existing relationship between vitamin D deficiency and thyroid gland disorders had been reported in the pathogenesis of keratoconus [15, 16]. Many studies had attempted to determine whether low vitamin D levels were associated with the presence and severity of KC [12] and whether they are caused by an isolated inflammatory action [5] or due to VDR and thyroid receptor interaction [17, 18].

The aim of this study was to detect the serum level of thyroid hormones, serum vitamin D and VDR polymorphism in KC patients and to identify any association between vitamin D deficiency and thyroid disorders in those patients

## Subjects and methods

This prospective, observational, cross sectional study was conducted at Mansoura ophthalmic center, faculty of medicine, Mansoura University in the period from March 2021 to September 2021. It included 177 keratoconus patients without any previously diagnosed thyroid disorders in comparison with 85 healthy controls with normal cornea and without any systemic diseases. This sample of individuals was selected from population in Dakahlia Governorate in Delta, Lower Egypt. The control group was selected from candidates of refractive surgery with normal topography that were free from post-Lasik ectasia for at least six months postoperatively. This study adhered to the tenets of Declaration of Helsinki and was approved by Mansoura faculty of medicine Institutional Review Board (code No R.21.01.1157.R1) and was registered on www.clinicaltrials.gov (NCT05073601). Each patient provided a written informed consent.

Diagnosis of keratoconus was done by a cornea specialist depending on slit lamp biomicroscopy findings of localized corneal thinning and ectasia which was confirmed by Pentacam (Oculus Optikgeräte GmbH, Wetzlar, Germany) examination. For each patient flat (K1), steep (K2), and maximum (Kmax) simulated keratometric readings, and corneal pachymetry were recorded. Patients who could not provide the informed consent or the necessary samples for any reason, patients who had any ophthalmic pathology other than KC, and those with systemic disorders were excluded from the study.

### Sample collection

A 5 ml venous blood was withdrawn from all subjects included in the study; 2 ml on EDTA for genetic study and 3 ml on a plain tube for thyroid hormones and serum 25-(OH) vitamin D measurement. The blood samples for hormonal assessment were centrifuged then serum samples were stored at −20 °C till the analysis time

### Laboratory investigations

To all patients and controls, quantitative measurements of thyroid hormones including thyroid stimulating hormone (TSH), free triiodothyronine (FT3) & free tetraiodothyronine (FT4) were done using enzyme linked immunosorbent assay (ELISA) kits supplied by CTK Biotech (San Diego, CA, USA). Serum 25-OH vitamin D was measured using ELISA kits supplied by MyBioSource (San Diego, CA, USA). Serum 25-OH vitamin D was classified based on 2012 American Endocrine Society guidelines [19]. Interpretation of the common patterns of thyroid function tests were as follows: Euthyroidism (TSH, FT3 and FT4 are normal), subclinical hypothyroidism (high TSH with normal FT3 and FT4), overt hypothyroidism (high TSH and low FT3 and/or FT4), subclinical hyperthyroidism (low TSH with normal FT3 and FT4) and overt hyperthyroidism (low TSH and high FT3 and/or FT4) [20].

Vitamin D receptors polymorphisms were done as follows: DNA extraction from the whole blood using Gene Jet gene DNA Purification kits supplied by Thermo Scientific (Rath Business Park, Dublin, Ireland), then the three VDR polymorphisms were tested [Taq I (rs731236), Apa I (rs7975232) and Bsm I (rs1544410)] using polymerase chain reaction-restriction fragment length polymorphism (PCR-RFLP). The amplification of genomic DNA was done using certain primers as follows:

Taq-I: Forward: 5′-CGGGGAGTATGAAGGACAAA-3′

Reverse: 5′-CCATCTCTCAGGCTCC AAAG-3′

Apa-I: Forward: 5′-CTAGGTCTGG ATCCTAAATGCA-3′

Reverse: 5′-TTAGGTTGGACAGGAGAGAGAA-3′

Bsm-I: Forward: 5′-CTAGGTCTGG ATCCTAAATGCA-3′

Reverse: 5′-TTAGGTTGGACAGGA GAGAGAA-3′

Genetic features of studied single nucleotide polymorphism were validated by the National Center for Biotechnology Information.

For all PCR reactions, 50 µL containing 10 µg of genomic DNA, 25 pmoL of each primer, 25 µL of Dream Taq Green PCR Master Mix (including 2.5 units of Taq Polymerase), and 18 µL of double distilled water were used. PCR was performed by an initial denaturation cycle at 95 °C for 3 min, annealing for 30 cycles at 95 °C for 30 s, primer specific temperatures used for 30 s (TaqI 61 °C, ApaI 72 °C and 58.5 °C for BsmI) and a temperature of 72 °C for 30 s, and the final step of extension at 72 °C for 5 min [21].

**Fig. 1:**
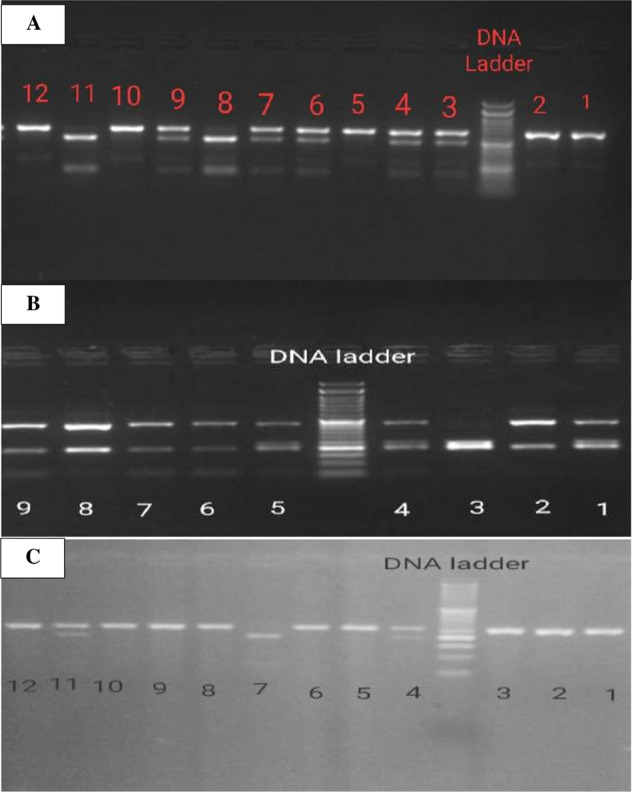
Agarose gel electrophoresis showing the PCR-RFLP of VDR genotypes. **A** Apa I genotypes. Lanes 3, 4, 6, 7, and 9 represent Aa genotype. Lanes 5, 10, and 12 represent AA genotype. Lanes 8 and 11 represent aa genotype. **B** Taq I genotypes: Lanes 1, 4, and 5 represent Tt genotype. Lanes 2, 6, 7, and 9 represent TT genotype. Lane 3 represents tt genotype. **C** Bsm I genotypes: Lanes 1, 2, 3, 5, 6, 8, 9, 10, and 12 represent BB genotype. Lanes 4 and 11 represent Bb genotype. Lane 7 represents bb genotype.

The PCR products were then digested by restriction enzymes (Taq I, Apa I, and Bsm I) supplied by Thermo Scientific. The enzyme-specific restriction reactions were performed according to the insert supplied with the kits. Then, the separation of DNA fragments was done using 2% agarose gel electrophoresis and visualized under UV light (Fig. 1A–C).

### Statistical analysis

Sample size is calculated using online sample size calculator available at (https://www.openepi.com/SampleSize/SSMean.htm). The collected data was analyzed using the Statistical Package of Social Science (SPSS) program for Windows (SPSS, Inc, Chicago, IL) version 22. Kolmogorov-Smirnov test was used to test data normality. Quantitative data were presented as median (range) as they were not normally distributed while qualitative data were described using number and percent. Mann-Whitney test was used to compare two medians. Chi-square test or Fisher’s exact test were used to test the association between categorical variables. Chi-squared test was used to determine the deviations from Hardy–Weinberg equilibrium (HWE) expectations. A logistic regression model using a backward stepwise method was used for the significant parameters. Odds ratio (OR) and 95% confidence interval (CI) were calculated. The results were considered to be significant when the probability of error is less than 5% (*p* < 0.05).

## Results

This cross-sectional study included 177 keratoconus patients compared to 85 age and sex-matched healthy subjects with normal cornea which were enrolled as a control group. Demographic and topographic parameters of the included subjects showed a significant increase in K1, K2 and K max in keratoconus group compared to controls, and a significant reduction in pachymetry in keratoconus group compared to controls. Laboratory investigations showed a significant increase in TSH level and FT4 (*P* = 0.001 and <0.001) respectively and the frequency of thyroid disorders was statistically significant higher in KC group. There was a significant decrease in serum 25(OH) vitamin D level and significant increase in vitamin D insufficiency and deficiency in KC group compared to control group (Table 1).

**Table 1:** Demographic, topographic, and laboratory data of studied subjects.

		KC group (177 subjects)	Control group (85 subjects)	*P* value
Age		29.7 ± 10.17	31.03 ± 10.12	0.320
Gender (no.): (male/female)		48/93	39/46	0.811
K1 (D)		48.3 (42.8–78.0)	42.3 (41.4–43.5)	**<0.001**
K2 (D)		51.5 (44.4–81.3)	43.1 (42.1–45.0)	**<0.001**
Kmax (D)		57.2 (46.3–89.3)	44.6 (43.1–46.0)	**<0.001**
Pachymetry (µm)		457.0 (211.0–540.0)	514.0 (489.0–560.0)	**<0.001**
TSH (µIU/L)		2.3 (0.04–14.0) (N range 0.4–4UIu/L)	1.5 (0.08–7.5)	**0.001**
FT4 (ng/dl)		1.43 (0.5–12.0) (N range 0.58–2.46 ng/dl)	1.0 (0.8–1.8)	**<0.001**
FT3 (pg/ml)		2.1 (0.99–11.2) (N range 1.4–4.2 pg/dl)	1.9 (1.2–3.9)	0.831
Thyroid state	euthyroid	138 (78.0%)	81 (95.3%)	**0.004**
	Subclinical hypothyroidism	15 (8.5%)	1 (1.2%)	
	Overt hypothyroidism	3 (1.7%)	0 (0.0%)	
	Subclinical hyperthyroidism	7 (4.0%)	3 (3.5%)	
	Overt hyperthyroidism	14 (7.9%)	0 (0.0%)	
25(OH)vitamin D(ng/ml)	10.6 (6.9–62.0)	31.0 (8.5–61.0)	**<0.001**	
25(OH) vitamin D	Sufficiency (>30 ng/ml)	19 (10.7%)	43 (50.6%)	**<0.001**
	Insufficiency (21–30 ng/ml	95 (53.7%)	31 (36.5%)	
	Deficiency (<20 ng/ml)	63 (35.6%)	11 (12.9%)	

Reference for thyroid hormones according to the insert supplied with the kit.

Reference for the vitamin D level is according to international standard.

The bold numbers refer to the statistically significant values.

*KC* keratoconus, *K1* flat keratometry, *K2* steep keratometry, *Kmax* maximum Keratometry, *D* diopters, *TSH*  thyroid-stimulating hormone, *FT4* free tetraiodothyronine, *FT3* free triiodothyronine, *OH* hydroxy.

Applying Hardy Weinberg equation revealed that TaqI, ApaI and BsmI genotypes in two groups are in HWE. As regards the distribution of VDR genotypes Taq 1 was significantly associated with KC. The tt gene variant alleles were of higher statistically significant distribution in KC patients when compared to controls indicating that tt genotype has higher risk to develop KC (*P* = 0.001, OR = 3.459). On the other hand, there was no statistically significant association between the APA 1 or BSM1 and KC (Table 2).

**Table 2:** Distribution of VDR genotypes and gene variant alleles in KC patients versus control group.

KC patients (*n* = 177) *n* (%)			Control (*n* = 85) *n* (%)	Relative risk of KC			
				OR		95% CI	*P* value
Taq I	TT	37 (20.9%)	32 (37.6%)	**1**	**-**	**-**	**R**
	Tt	80 (45.2%)	38 (44.7%)	1.820	0.988	3.353	0.054
	tt	60 (33.9%)	15 (17.6%)	**3.459**	**1.654**	**7.233**	**0.001**
	Tt + tt	140 (79.1%)	53 (62.4%)	**2.284**	**1.293**	**4.035**	**0.004**
	T	154 (43.5%)	102 (60.0%)	**1.948**	**1.343**	**2.825**	**<0.001**
	t	200 (56.5%)	68 (40.0%)				
Apa I	AA	64 (36.2%)	26 (30.6%)	**1**	**-**	**-**	**R**
	Aa	85 (48.0%)	42 (49.4%)	0.822	0.457	1.478	0.513
	aa	28 (15.8%)	17 (20.0%)	0.669	0.314	1.424	0.297
	Aa + aa	113 (63.8%)	59 (69.4%)	0.778	0.447	1.354	0.374
	A	213 (60.2%)	94 (55.3%)	0.818	0.565	1.185	0.289
	a	141 (39.8%)	76 (44.7%)				
Bsm I	BB	61 (34.5%)	23 (27.1%)	**1**	**-**	**-**	**R**
	Bb	75 (42.4%)	42 (49.4%)	0.673	0.365	1.239	0.204
	bb	41 (23.1%)	20 (23.5%)	0.773	0.376	1.585	0.482
	BB + bb	116 (65.5%)	62 (72.9%)	0.705	0.398	1.247	0.230
	B	197 (55.6%)	88 (51.8%)	0.855	0.592	1.234	0.403
	b	157 (44.4%)	82 (48.2%)				

The bold numbers refer to the statistically significant values.

*VDR* vitamin D receptors, *KC* keratoconus, *OR* odds ratio, *CI* Confidence interval, *R* reference.

Comparison of vitamin D level among the studied VDR genotypes in KC patients reported a significantly higher serum 25(OH) vitamin D level in TT genotype, while insufficient level was more common in Tt genotype. In addition, a deficient serum 25(OH) vitamin D level was predominant in tt genotype (Table 3).

**Table 3:** Comparison of Vitamin D level among studied VDR genotypes in KC patients.

Parameter		Taq I genotypes			*P* value
		TT (*n* = 37patients)	Tt (*n* = 80 patients)	tt (*n* = 60 patients)	
Vitamin D level (ng/ml)	Median (Min–Max)	19.3 (10.0–62.0)	10.2 (7.6–51.0)	9.5 (6.9–42.0)	**<0.001**
Vitamin D groups	Sufficiency *n* (%)	10 (27.0%)	5 (6.2%)	4 (6.7%)	**<0.001**
	Insufficiency *n* (%)	27 (73.0%)	48 (60.0%)	20 (33.3%)	
	Deficiency *n* (%)	0 (0.0%)	27 (33.8%)	36 (60.0%)	

Parameter		Apa I genotypes			*P* value
		AA (*n* = 64)	Aa (*n* = 85)	aa (*n* = 28)	
Vitamin D level (ng/ml)	Median (Min–Max)	12.8 (7.6–62.0)	10.4 (7.5–59.0)	9.9 (6.9–51.0)	0.243
Vitamin D groups	Sufficiency *n* (%)	11 (17.2%)	7 (8.2%)	1 (3.6%)	0.072
	Insufficiency *n* (%)	35 (54.7%)	48 (56.5%)	12 (42.8%)	
	Deficiency *n* (%)	18 (28.1%)	30 (35.3%)	15 (53.6%)	

Parameter		Bsm I genotypes			*P* value
		BB (*n* = 61)	Bb (*n* = 75)	bb (*n* = 41)	
Vitamin D level (ng/ml)	Median (Min–Max)	12.0 (6.9–62.0)	10.0 (7.3–59.0)	12.8 (7.6–42.0)	0.567
Vitamin D groups	Sufficiency *n* (%)	4 (6.6%)	8 (10.7%)	7 (17.1%)	0.441
	Insufficiency *n* (%)	37 (60.7%)	38 (50.7%)	20 (48.8%)	
	Deficiency *n* (%)	20 (32.7%)	29 (38.6%)	14 (34.1%)	

The bold numbers refer to the statistically significant values.

*VDR* vitamin D receptors, *KC* keratoconus.

Univariate analysis was done to study risk factors for KC development. The following parameters: TSH, T4, Hypothyroidism, Hyperthyroidism, vitamin D level, insufficient and deficient vitamin D, and tt genotype of Taq1 were found to be statistically significant (*P* < 0.05).

Logistic regression analysis was conducted in KC group, using the above significant covariates, TSH, T4, hypothyroidism, insufficient and deficient vitamin D were predicted to be risk factors for the disease. On the other hand, sufficient vitamin D was a significant protective factor (Table 4).

**Table 4:** Logistic regression analysis in keratoconus group.

		Multivariate analysis			
		*p*	OR	95% CI	
TSH		<0.001	2.173	1.603	2.946
T4		<0.001	61.95	24.145	77.06
Thyroid disorder	Hypothyroidism vs normal	0.044	8.474	1.062	67.63
	Hyperthyroidism vs normal	0.086	3.142	0.852	11.58
Vitamin D		<0.001	0.943	0.915	0.917
Vitamin D group	Insufficient vs sufficient	<0.001	5.847	2.936	11.643
	Deficient vs sufficient	<0.001	12.314	5.283	28.704
Taq I	tt vs TT	0.237	1.910	0.654	5.576

*KC* keratoconus, *OR* odds ratio, *CI* Confidence interval, *P* *p* value.

## Discussion

This study aimed to identify any association between vitamin D deficiency and thyroid disorders in Kc patients as both of them are potential factors that were recently linked to KC development [8, 22, 23].

Regarding thyroid dysfunction, this study reported a statistically significant higher incidence of thyroid dysfunction with more predominance of hypothyroidism in KC patients in comparison to healthy controls. This was in agreement with previous studies which reported that hypothyroidism may play a role in the exaggeration or even initiation of KC [8, 24–26]. Others have demonstrated that thyroxin in tear fluid may have a possible role in KC development [24]. The relationship between KC and thyroid disorders is not yet well settled. Karabulut et al. [25] showed that patients with Grave’s disease have significantly altered corneal biomechanical properties, while Gatzioufas et al. [11, 26] found that hypothyroidism may induce corneal topographical and biomechanical changes or even exacerbate KC resulting in acute corneal hydrops. Further studies are needed to determine whether the increase or the decrease in thyroid hormones levels is more associated with KC development and progression.

Previous studies [15, 16] reported the involvement of Vitamin D and thyroid dysfunctions in KC development as they both work on receptor belonging to steroid/thyroid receptor family. The present study revealed a significant decrease in serum vitamin D level in KC patients than the control group. In addition, there was a significant increase in both vitamin D insufficiency and vitamin D deficiency (53.7% and 35.6% in KC group compared to 36.5% and 12.9% in control group respectively). This finding goes in agreement with the study of Akkaya and Ulusoy [22] who reported a lower serum vitamin D level in KC patients when compared to the healthy controls and this was linked to the inflammatory and immunological nature of the KC. This was again supported by Ghanavati et al. [18] who demonstrated that inflammatory, apoptotic, or oxidative mechanisms may cause ocular surface affection due to vitamin D deficiency. Moreover, researchers found that decreased vitamin D level significantly increased non progressive KC probability by 1.23 and progressive KC probability by 1.29 more than the control group. They concluded that evaluating serum vitamin D levels in KC patients at the onset and the follow-up examinations may predict the disease course [23].

In this study, we also evaluated the association between the most common VDR polymorphisms: TaqI (rs731236), BsmI (rs1544410) and ApaI (rs7975232) with KC. To the best of our knowledge this is the first research to study this association. It was found that Taq1 polymorphism was associated with an increased risk of KC. The tt genotype had a higher risk to develop KC as t allele was more prevalent in KC patients (56.5%) while T allele was shown to have a protective effect against the disease (60% in control group compared to 43.5% in KC group). On the other hand, APA 1 and BSM1 were silent polymorphisms not associated with an increased risk of KC. Assessment of vitamin D level among studied VDR genotypes in KC patients revealed a significantly higher serum vitamin D level in TT genotype and insufficient serum vitamin D level in Tt genotype, while deficient serum vitamin D level was predominant in tt genotype.

In our study, Logistic regression analysis for prediction of keratoconus using the multivariate analysis revealed that TSH, T4, hypothyroidism, insufficient and deficient vitamin D levels were significantly associated with keratoconus.

One of the Limitations of our study is that we didn’t correlate the vitamin D level to keratoconus grade and the state of progression. Future studies are needed to assess this relationship and to confirm the effect of vitamin D supplementation on keratoconus stability. In addition, Assessment of parathyroid hormone is also needed to study its effect on thyroid hormones and vitamin D which may have a direct role in KC development.

## Summary table

### What was known before

- The exact pathophysiology of keratoconus is not well understood. Thyroid disorders and vitamin D may play a role in disease development

### What this study adds

- Thyroid dysfunction and vitamin D deficiency are significantly associated with keratoconus. vitamin D receptor polymorphism is associated with keratoconus development. Taq1 gene and its tt alleles were reported as high risk factors for development of keratoconus.

## Data Availability

Derived data supporting the findings of this study are available from the corresponding author on request.
